# The TRPM3 ion channel mediates nociception but not itch evoked by endogenous pruritogenic mediators

**DOI:** 10.1016/j.bcp.2020.114310

**Published:** 2020-10-29

**Authors:** Balázs Kelemen, Silvia Pinto, Nawoo Kim, Erika Lisztes, Martin Hanyicska, Anita Vládar, Attila Oláh, Zsófia Pénzes, Brian Shu, Joris Vriens, Tamás Bíró, Tibor Rohács, Thomas Voets, Balázs István Tóth

**Affiliations:** a Department of Physiology, Faculty of Medicine, University of Debrecen, Debrecen, Hungary; b Laboratory of Ion Channel Research (VIB-KU Leuven Center for Brain & Disease Research) Department of Cellular and Molecular Medicine, KU Leuven, Leuven, Belgium; c Department of Immunology, Faculty of Medicine, University of Debrecen, Debrecen, Hungary; d Doctoral School of Molecular Medicine, University of Debrecen, Debrecen, Hungary; e Department of Pharmacology, Physiology and Neuroscience, Rutgers New Jersey Medical School, Newark, NJ, United States; f Laboratory of Endometrium, Endometriosis and Reproductive Medicine, Department of Development and Regeneration, KU Leuven, Leuven, Belgium

**Keywords:** Nociception, Itch, TRP channels, TRPM3, Cheek model, Endogenous pruritogens

## Abstract

During the molecular transduction of itch, the stimulation of pruriceptors on sensory fibers leads to the activation or sensitization of ion channels, which results in a consequent depolarization of the neurons. These ion channels mostly belong to the transient receptor potential (TRP) channels, which are involved in nociception and thermosensation. In particular, TRPV1 and TRPA1 were described in the transduction of both thermal nociception as well as histaminergic and non-histaminergic itch. The thermosensitive TRPM3 plays an indispensable role in heat nociception together with TRPV1 and TRPA1. However, the role of TRPM3 in the development of pruritus has not been studied yet. Therefore, in this study we aimed at investigating the potential role of TRPM3 in the transduction of pruritus and pain by investigating itch- and nociception-related behavior of *Trpm3*^+*/*+^ and *Trpm3*^−*/*−^ mice, and by studying the activation of somatosensory neurons isolated from trigeminal ganglia upon application of algogenic and pruritogenic substances. Activators of TRPM3 evoked only nocifensive responses, but not itch in *Trpm3*^+*/*+^ animals, and these nocifensive responses were abolished in the *Trpm3*^−*/*−^ strain. Histamine and endogenous non-histaminergic pruritogens induced itch in both *Trpm3*^+*/*+^ and *Trpm3*^−*/*−^ mice to a similar extent. Genetic deletion or pharmacological blockade diminished TRPM3 mediated Ca^2+^ responses of sensory neurons, but did not affect responses evoked by pruritogenic substances. Our results demonstrate that, in contrast to other thermosensitive TRP channels, TRPM3 selectively mediates nociception, but not itch sensation, and suggest that TRPM3 is a promising candidate to selectively target pain sensation.

## 1. Introduction

Although pain and itch are associated with clearly different subjective sensations, their general definitions emphasize common features: both can be described as an unpleasant sensation associated with protective somatosensory responses [1]. Our knowledge about how the somatosensory system manages pain and itch sensation have advanced a lot in the last two decades, but there are still several controversies and open questions regarding the relationship between nociception and pruriception, as well as the molecular mechanisms underlying their sensory transduction. Nowadays the “selectivity theory of itch” is increasingly accepted, which states that a subpopulation of nociceptive fibers innervating the skin can be activated by various pruritogenic substances, and that the selective activation of these fibers results in itch sensation, whereas the more general activation of nociceptors evokes pain [2]. Although serious efforts were made to reveal molecular markers of itch-specific neurons [3–7], the molecular mechanisms of the sensory transduction of painful and pruritic stimuli seems to overlap [8–13].

The activation of nociceptors is initiated by the opening of nociceptive ion channels, including thermosensitive transient receptor potential (TRP) channels, acid sensing ion channels (ASICs) [14] or P2X ionotropic purinoreceptors [15–17], which results in depolarization and consequent discharge of the nociceptive neurons. These ion channels can be directly activated by painful stimuli (extreme temperatures, acidosis or pain-evoking chemical ligands) or indirectly, via intracellular signaling pathways initiated by algogenic or inflammatory signals [18]. Thermosensitive TRP channels play an especially important role as multimodal integrators of various painful stimuli [19]. For example, TRP vanilloid 1 (TRPV1), probably the most studied thermo-TRP channel, is directly activated by noxious heat, acidosis or its potent chemical ligand capsaicin (each evoking pain) and can also be sensitized by inflammatory mediators [20–25]. Beyond TRPV1, TRPA1, TRPM8 and the recently characterized TRPM3 also play important roles in the transduction of thermal, chemical and inflammatory pain in somatosensory neurons [19,26–32]. Although the activation of pruriceptive (itch sensitive) sensory neurons is also related to the activation of ion channels partially overlapping with the nociceptor channels, they are typically activated via an indirect way: pruritic ligands bind to their metabotropic receptors thereby activating the pruritic channels via downstream signaling pathways [8,9]. A particular role of the heat-pain mediating TRPV1 and TRPA1 was also described in the transduction of histaminergic and non-histaminergic itch [11,12,33–36].

TRPM3 was recently identified as a heat sensitive TRP channel expressed by somatosensory neurons of the trigeminal and dorsal root ganglia (TGs and DRGs). Its chemical activators evoke nocieption, and the channel also plays a role in inflammatory thermal hyperalgesia [30,32,37]. Together with the above mentioned TRPV1 and TRPA1, it has an essential contribution to the heat-induced nociception [38]. However, in contrast to TRPV1 and TRPA1, the potential role of TRPM3 in itch was not investigated yet. Therefore, in this study we aimed at describing its role in nociception and itch sensation by comparing itch-related and nocifensive behavior in wild type (*Trpm3*^+*/*+^) and TRPM3 deficient (*Trpm3*^−*/*−^) mice *in vivo*, and by exploring the responses of their sensory neurons to algogenic TRP ligands and well known endogenous pruritogens *in vitro*.

## 2. Materials and methods

### 2.1. Animals

8–14 week-old wild-type (*Trpm3*^+*/*+^, from Janvier labs, Le Genest Saint Isle, France) and TRPM3 deficient (*Trpm3*^−/−^, established in our laboratory at KU Leuven (Leuven, Belgium) as described in our previous publication) [32] C57BL/6J mice weighted 20–30 g were used in all experiments. Only male mice were used to study itch and nocifensive behavior in the cheek assay to eliminate any potential influence of the estrus cycle on the scratching behavior, and male and female mice were used in other experiments. Mice were housed in a conventional animal facility at constant 21 °C in a 12-h light–dark cycle with unrestricted access to food and water. All animals were drug and test naïve prior being recruited to experiments. All experiments using animals were carried out in accordance with the European Union Community Council guidelines and approved by the KU Leuven Ethical Committee for Animal Experimentation under project number P021/2018 or by the Institutional Animal Care and Use Committee at Rutgers New Jersey Medical School.

### 2.2. Behavior assays

#### 2.2.1. Cheek-assay

The itch-induced scratching and nociception-related behavior was selectively assessed using the cheek model paradigm [39]. Mice were habituated to a plexiglass recording chamber and the observation room for 30 min, once daily during the week before testing. The fur of the affected cheek was shaved a day in advance of the experiment. The mouse was held tightly by the experimenter and the fur was shaved with a single movement using a small electric hair clipper (Aesculap Isis from Aesculap Suhl GmbH, Shul, Germany), carefully avoiding the whiskers and not hurting the skin. On the day of the experiment, mice were randomly allocated into experimental groups by a person who was not involved in the further investigation. Mice were placed into the plexiglass recording chamber and video recording was started. 10 min later, investigated compounds were applied s.c. via microinjections into the cheek using a 30G needle attached to a 1 ml insulin syringe. The applied doses were selected based on previous literature data and tested in pilot experiments. The following compounds were applied: 10 μg pregnenolone sulfate (PregS), 10 μg capsaicin (Caps), 5 μg CIM0216 (all from Tocris, Bristol, UK), 50 μg histamine (Hist), 10 μg serotonin (5-HT) and 150 ng endothelin-1 (ET-1) (all pruritogens from Sigma-Aldrich, St. Louis, MO, USA) each dissolved in 10 μl of Ca^2+^- and Mg^2+^-free phosphate buffered saline (PBS) with 7% of Tween-80 (Sigma-Aldrich) and injected as a 10 μl volume [39]. Then, mice were placed back into the recording chamber, the experimenter left the room and spontaneous behavior was captured for an additional 30 min after the injection. The amount of time each mouse spent scratching, the number of scratch bouts and the number of wipes on the injected site were quantified over the course of a 30-min period following the injection. One bout of scratching was defined as an episode in which a mouse lifted its hind paw and scratched continuously for any length of time, until the paw was returned to the floor or to its mouth. During treatment and behavioral scoring, investigators were blinded for genotype and treatment.

#### 2.2.2. Calculation of scratch ratio (R_scratch_)

Based on the results of the cheek assay, the pruritogenic and algogenic nature of the compounds applied was characterized by calculating a novel parameter, the scratch ratio (R_scratch_), defined as: R_scratch_ = N_scratch_/(N_scratch_ + N_wipe_), where N_scratch_ and N_wipe_ are the number of scratches and wipes, respectively, detected during the observation. Value of R_scratch_ can vary between 0 and 1 where 0 indicates pure nociception related responses (wipes) without any scratching, and 1 represents exclusively itch-related behavioral responses. Values in the middle range of the scale are characteristic for neutral compounds inducing neither significant nociception nor itch or for compounds inducing similarly frequent nocifensive and itch responses.

#### 2.2.3. Nape-assay

In order to further assess the potential role of TRPM3 in mediating itch in different regions of the skin, pruritogenic compounds were also injected into the nape of *Trpm3*^+*/*+^ and *Trpm3*^−*/*−^ mice. The animals were acclimated to the test chamber the day before the experiment for 1 h, and the nape of the neck was shaved. The experimenter restrained the mouse with one hand and carefully shaved the nape area behind the ears using small electronic clippers (PepPet, Guangdong, China). Care was taken to avoid skin and ear injury. Mice were allowed to acclimatize for 10 min before the injection took place on the day of the experiment. Pruritogenic compounds were injected s.c. into the nape, and the mouse was immediately placed back into the chamber, the experimenter left the room, and scratching behavior was video recorded for 30 min. As pruritogenic compounds, 200 μg Hist, 10 μg 5-HT, and 250 ng ET-1 dissolved in 50 μl PBS were injected. The applied doses and injection volume were selected corresponding to previous literature data and tested in pilot experiments. The records were analyzed by experimenters blinded to genotype and compound injected.

### 2.3. Culturing and isolation of sensory neurons

Sensory neurons of trigeminal ganglia (TGs) were obtained from 8 to 12 week-old *Trpm3*^+*/*+^ and *Trpm3*^−*/*−^ mice, as described before [32,37]. Briefly, mice were euthanized by CO_2_, TGs were isolated and digested with collagenase (2 mg/ml) and dispase (2,5 mg/ml) (both from Invitrogen/Thermo Fisher Scientific, Waltham, MA, USA). Suspension of sensory neurons was seeded on laminin (100 μg/ml) and poly-L-ornithine HBr (500 μg/ml) (both from Sigma Aldrich) coated glass bottom culture dishes (MatTek, Ashland, MA, USA) and cultured in Neurobasal medium supplemented with 2% B-27 supplement, 2 mM L-glutamine, 100 μg/ml penicillin/streptomycin, 2 ng/ml glial cell line-derived neurotrophic factor (GNDF) (all from Invitrogen/Thermo Fisher Scientific) and 10 ng/ml NT-4 (PeproTech, London, UK) at 37 °C in 5% CO_2_ containing humidified atmosphere. Neurons were used for experiments within 24 to 36 hrs following isolation.

### 2.4. Fluorescent measurements of intracellular Ca^2+^ concentration

To measure the cytoplasmic Ca^2+^ concentration in individual sensory neurons, we used microscope-based calcium imaging systems. On the day after the isolation, TG neurons were loaded with acetoxymethyl ester-conjugated fluorescent Ca^2+^ indicators dissolved in culturing medium. 2 μM Fura-2-AM (Invitrogen/Thermo Fisher Scientific) was used in experiments comparing the responses of sensory neurons from *Trpm3*^+*/*+^ and *Trpm3*^−*/*−^ animals, and 2 μM Fluo-4-AM (Invitrogen/Thermo Fisher Scientific) was applied when investigating the effect of pharmacological inhibition of TRPM3. Both dyes possess a Kd value for Ca^2+^ in submicromolar range, and are therefore equally suitable to detect relevant changes in cytoplasmic Ca^2+^ concentration. Fura-2-loaded cells were placed on the stage of a Nikon fluorescent microscope and captured with constant setting every 1 s (λ1_EX_:340 nm, λ2_EX_: 380 nm and λ_EM_: 505 nm) and data were obtained as the ratio of the fluorescence measured at 340 and 380 nm excitation wavelengths (F_340_/F_380_). Fluo-4 loaded cells were placed on the stage of a Zeiss LSM 5 Live confocal microscope and captured at λ1_EX_:488 and λ_EM_: 516 once every second and data were presented as F_1_/F_0_, where F_0_ is the average fluorescence of the baseline (before the first compound application) and F_1_ is the actual fluorescence. During the measurements, cells were continuously perfused with Ca^2+^-buffer (150 mM NaCl, 5 mM KCl, 1 mM MgCl_2_×6H_2_O, 2 mM CaCl_2_×2H_2_O, 10 mM glucose xH_2_O, 10 mM HEPES, pH 7.4 (all from Sigma-Aldrich)) and different compounds were applied via the perfusion. All experiments were performed at room temperature (21–22 °C).

### 2.5. Materials

The endogenous TRPM3 agonist PregS, the TRPA1 agonist cinnamaldehyde (CA), the TRPV1 agonist Caps, and the exogenous TRPM3 agonist CIM0216 were obtained from Tocris Bioscience (Bristol, UK). The TRPM3 antagonist Isosakuranetin (Isok) was obtained from Carl Roth (Karlsruhe, Germany). The well-characterized pruritogen Hist was obtained from Sigma-Aldrich (Sigma-Aldrich, St. Louis, MO, USA); and the non-histaminergic endogenous pruritic mediators 5-HT and ET-1 were purchased from Abcam (Abcam, Cambridge, MA, USA) or Sigma Aldrich.

### 2.6. Data and statistical analysis

Origin 9.0 (OriginLab Corporation, Northampton, MA, USA) was used for analysis and data display for both *in vivo* and *in vitro* data. Statistical analysis was performed using IBM SPSS Statistics 22 software (IBM, Armonk, NY, USA). Kruskal-Wallis test was used to compare multiple groups. Whenever it reported significant differences, Mann-Whitney tests with Bonferroni adjustment were used as post hoc analysis for pairwise comparison (e.g. comparing the effect of several treatments within one genotype). The Mann-Whitney test was also applied to compare only two groups (e.g. *Trpm3*^+*/*+^ and *Trpm3*^−*/*−^ within a particular treatment). The distribution of various neurons between the different groups was compared using Chi-squared (Х^2^) test. In every case, P < 0.05 was regarded as showing significant differences between groups. If not mentioned otherwise, individual data were presented in scatterplots marking mean ± SD.

## 3. Results

### 3.1. TRPM3 agonists induced nociception but not itch in the mouse cheek model

To assess the role of TRPM3 in itch sensation and nociception, we investigated wild type (*Trpm3*^+/+^) and TRPM3-deficient (*Trpm3*^−/−^) C57/Bl6 mice [32] in the “cheek model” paradigm, which allows differentiation between behavioral responses related to nociception and pruriception [39].

**Fig. 1.**
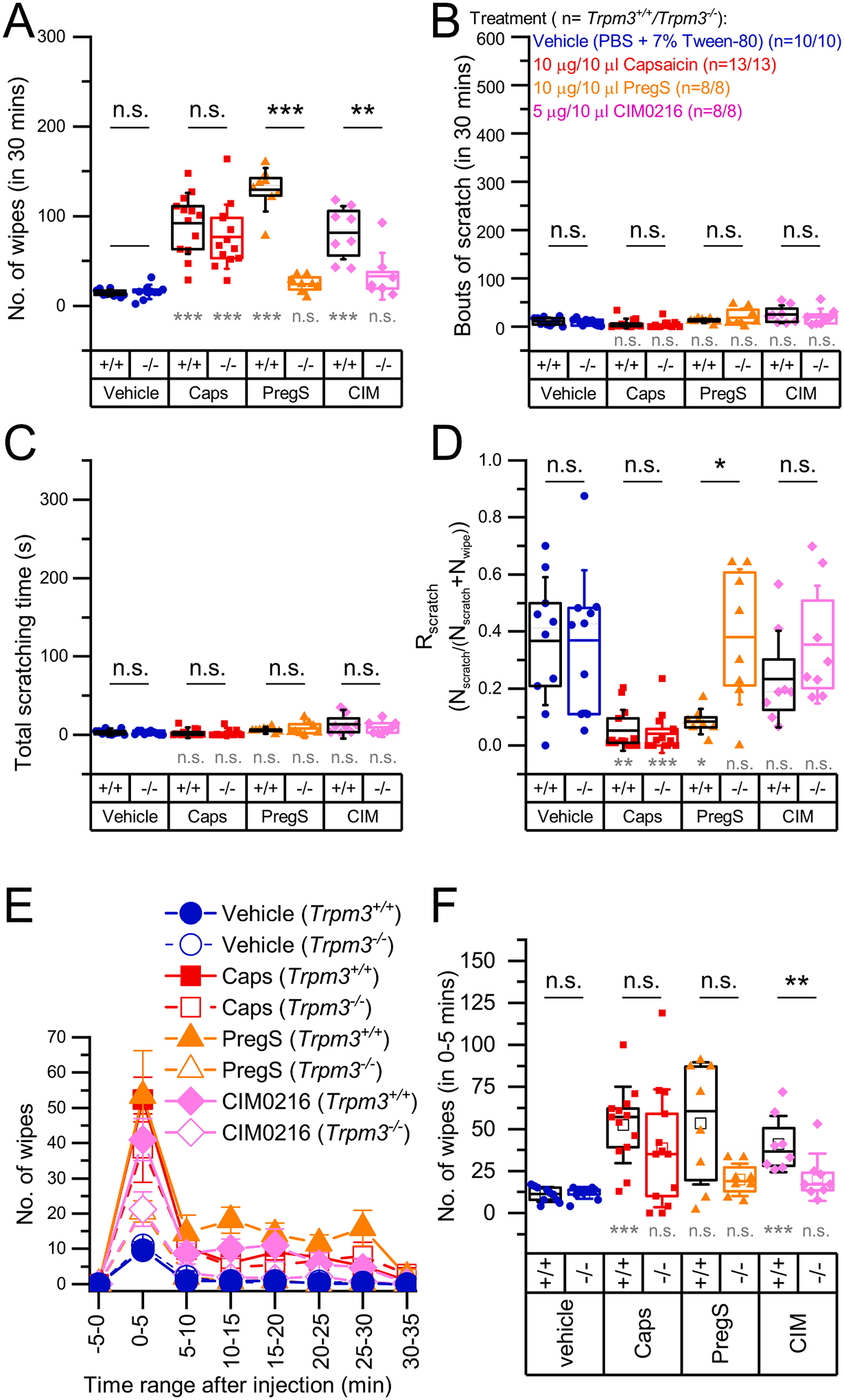
Pain and itch behavior induced by TRPM3 agonist and capsaicin in the mouse cheek model *Trpm3*^+/+^ and *Trpm3*^−*/*−^mice were injected in the cheek with the agonists indicated in the figure. (A) Number of wipes, (B) bouts of scratches, and (C) total time of scratching as determined within 30 min following the injection. (D) Scratch ratio (R_scratch_) calculated as described in the methods. (E) Mean time courses of the number of wipes after injecting the agonists in 5 min-long intervals within 35 min after injection. (F) Statistical comparison of the number of wipes in the 0–5 min interval, as indicated in in panel (E). Dots mark data from individual animals, boxes indicate 25–75 percentile, thick lines and thin lines in the boxes point the mean and median, respectively, whiskers show SD. n.s.: p > 0.05 (non-significant), *p < 0.05, **p < 0.01, ***p < 0.001 as compared either to the vehicle treated group within the same genotype using Kruskal-Wallis test with Bonferroni adjusted Mann-Whitney *U* test as post hoc analysis (grey marking) or compared between *Trpm3*^+*/*+^ and *Trpm3*^−*/*−^ within the same treatment, as indicated, using Mann-Whitney *U* test (black marking).

First, we injected the TRPM3 agonists PregS and CIM0216 to the cheek of the animals as described in the Methods section. The injection technique and behavioral analysis were optimized in preliminary experiments. As a negative control, we injected the same volume of the vehicle buffer and used the TRPV1 agonist Caps as a well-established algogenic substance to assess TRPM3-independent nocifensive responses. We found that TRPM3 agonists induced marked nocifensive responses in *Trpm3*^+*/*+^ animals compared to the vehicle control quantified by the number of wiping events on the injected cheek as described in the Materials and methods (Fig. 1A, Suppl. video file 1). These nocifensive responses were abolished in *Trpm3*^−*/*−^ animals, clearly indicating that PregS- and CIM0216-induced nociception is mediated by TRPM3 in the cheek. Caps also induced marked nocifensive behavior but it was not influenced by the deletion of *Trpm3*, demonstrating, as found earlier in other assays [32], that the effect of Caps is independent of TRPM3 in the cheek model. Importantly, similar to Caps, none of the TRPM3 agonists induced significant itch-related behavior in *Trpm3*^+/+^ and *Trpm3*^−/−^ animals, as assessed by the number of scratches and the total time spent with scratching (Fig. 1B–C).

To better characterize the quality of the sensory phenomena evoked by a particular compound, we introduced a new measure, “scratch ratio” (R_scratch_) as defined in the Methods. We found that TRPM3 agonists, as well as the TRPV1 agonist Caps behaved as algogenic substances in *Trpm3*^+/+^ animals i.e. they induced mainly nociception and hardly any itch. However, in *Trpm3*^−/−^ animals, PregS and CIM0216, but not Caps, were found to behave as neutral compounds, as R_scratch_ was near to 0.5 (Fig. 1D). Interestingly, PregS induced slightly more intense wiping in *Trpm3*^+/+^ animals than Caps (Fig. 1A), however detailed analysis of the observation suggested, that in the applied doses, Caps may induce more intense pain than PregS or CIM0216. For each agonist, the peak response was reached during the first 5 min, after which the responses decayed, and this decay was more pronounced in the capsaicin- than in the PregS-treated group (No. of wipes_0–5 min_ Caps vs. PregS: 52.38 ± 22.70 vs. 53.38 ± 36.24, U = 55.0, p = 0.860 and No. of wipes_10–15 min_ Caps vs. PregS: 6.00 ± 5.58 vs. 18.13 ± 10.52, U = 91.0, p = 0.003) (Fig. 1E). However, in the Caps treated group we observed additional nociception-related behavior including “tunneling” (where the animal digs a tunnel in the bedding while pressing the injected cheek to the bottom of the cage, Suppl. video file 2) and lethargy. Although, as previous studies assessing nociception in the cheek model, we restricted the quantitative analysis to the number of wipes (as a pre-defined measure), these earlier unreported patterns of behavior in the cheek assay may also indicate nociception as a form of counter irritation, analogue to pressing or rubbing a painful area aiming at alleviating pain. These signs of nociception were more typical in the Caps injected group in the later phase of the observation, and were less characteristic for the PregS-injected group.

### 3.2. TRPM3 is not involved in the sensation of itch induced by Hist or non-histaminergic pruritogens 5-HT and ET-1

**Fig. 2.**
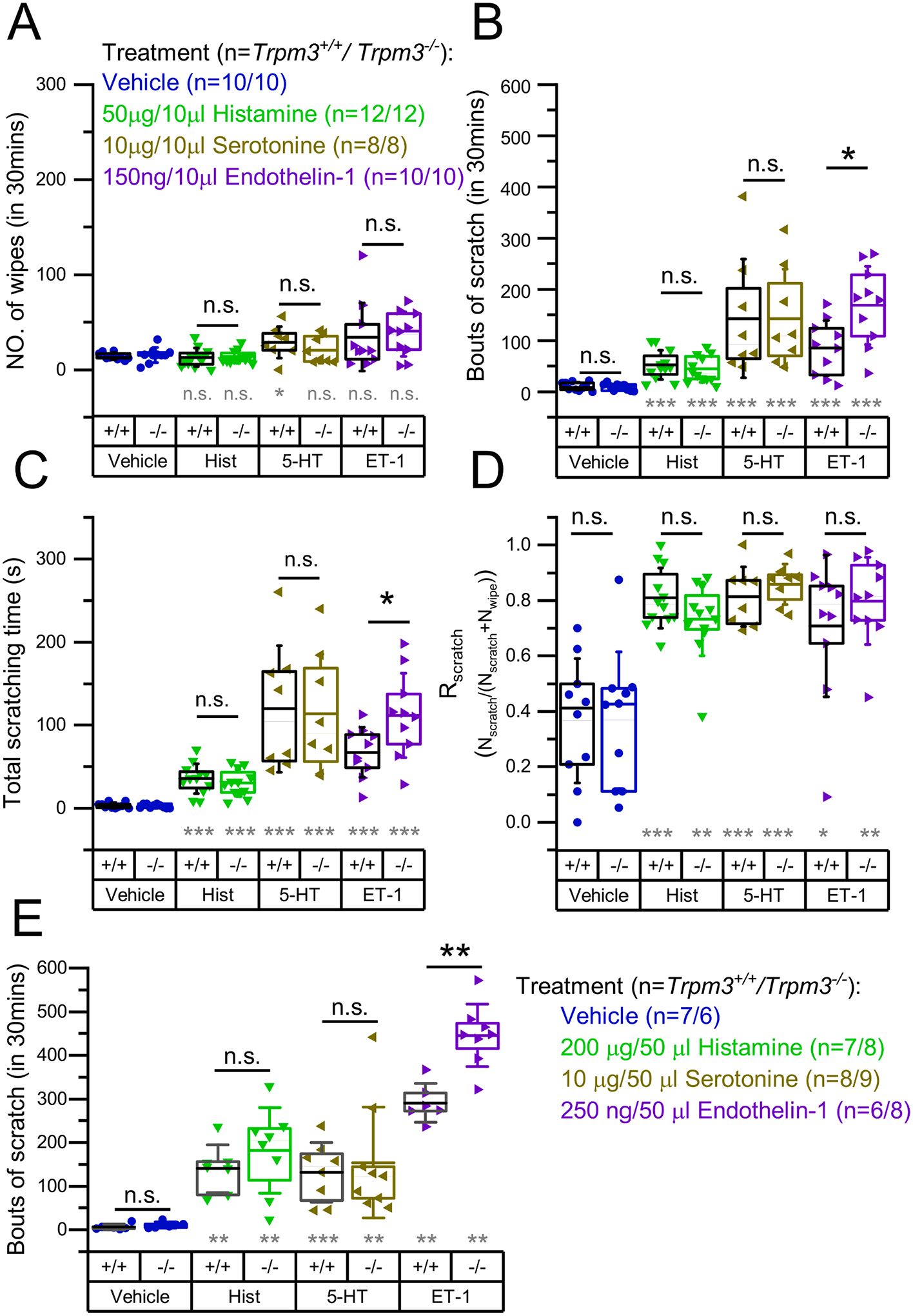
Quantification of the itch and pain related behavior induced by Hist, 5-HT, and ET-1 in *Trpm3*^+/+^ and *Trpm3*^−*/*−^ mice. *Trpm3*^+*/*+^ and *Trpm3*^−*/*−^ mice were injected in the cheek with the pruritogens indicated in the figure. (A) Number of wipes, (B) bouts of scratches, and (C) total time of scratching as determined within 30 min following the injection. (D) Scratch ratio (R_scratch_) calculated as described in the methods. (E) Bouts of scratches after injecting indicated compounds in the nape of *Trpm3*^+*/*+^ and *Trpm3*^−*/*−^ mice. Vehicle treated control groups presented in panel (A)–(D) are identical with those presented in Fig. 1A–D. Dots mark data from individual animals, boxes indicate 25–75 percentile, thick lines and thin lines in the boxes point the mean and median, respectively, whereas whiskers show SD. n.s.: p > 0.05 (non-significant), *p < 0.05, **p < 0.01, ***p < 0.001, between *Trpm3*^+*/*+^ and *Trpm3*^−*/*−^ within the same treatment, as indicated, using Mann-Whitney *U* test (black marking) or compared to the vehicle treated group within the same genotype using Kruskal-Wallis test with Bonferroni adjusted Mann-Whitney *U* test as post hoc analysis (grey marking).

Although direct activation of TRPM3, similar to TRPV1, resulted exclusively in nociception and not itch, these results cannot exclude that TRPM3 signaling can also contribute to the sensory transduction of pruritus, as has been described for TRPV1. Indeed, direct, general activation of TRPV1 is known to induce nociception and not itch, but TRPV1 expressed locally in pruriceptive sensory neurons takes part in the transduction of both histaminergic and some forms of non-histaminergic pruritus [9,11,31,40–42]. To investigate the role of TRPM3 in the sensory transduction of pruritus evoked by the endogenous mediators Hist, 5-HT and ET-1 (each known to evoke severe itch in both human and rodent models [43,44]), we tested these compounds in the cheek model paradigm in *Trpm3*^+*/*+^ and *Trpm3*^−*/*−^ mice. We found that Hist, 5-HT and ET-1 induced pronounced itch but hardly any nociception in *Trpm3*^+*/*+^ and *Trpm3*^−*/*−^ animals (Fig. 2A–C): the number of wipes detected was comparable to vehicle whereas the number of scratches and the time spent scratching were strongly elevated by each pruritogenic compound, irrespective of genotype. Most importantly, the number of scratches induced by the pruritogens was not decreased in the *Trpm3*^−*/*−^ strain compared to wild type animals. High R_scratch_ values also indicated that Hist, 5-HT, and ET-1 evoked a predominant pruritogenic and not algogenic effect in both strains (Fig. 2D). Interestingly, ET-1 induced itch was found to be significantly more intense in the *Trpm3*^−*/*−^ group than in the *Trpm3*^+*/*+^ group.

Since high R_scratch_ values indicated that Hist, 5-HT and ET-1 evoked mainly itch and hardly nociception, we also tested their effect injected in the nape of *Trpm3*^+*/*+^ and *Trpm3*^−*/*−^ animals. Although behavioral reactions after nape injection cannot clearly discriminate between itch and nociception (both induce similar scratching responses), known “pure” pruritogen compound-induced responses can be interpreted as signs of itch [39,44]. Studying the behavioral responses evoked by the aforementioned pruritogens in the nape, we aimed at investigating the role of TRPM3 in the innervation area of dorsal root ganglia (DRGs) to compare to the results of the cheek injections which affected the innervation area of the trigeminal ganglion (TG). We found that Hist and 5-HT evoked similarly intense pruritus in *Trpm3*^+*/*+^ and *Trpm3*^−*/*−^ mice, as we observed in case of cheek injection, as well (Fig. 2E). The ET-1 induced responses were also in line with the scratches evoked in the cheek model: *Trpm3*^−*/*−^ animals showed significantly stronger ET-1-induced itch responses than *Trpm3*^+*/*+^ mice.

### 3.3. Pruritogens activated trigeminal sensory neurons independently of TRPM3

**Fig. 3.**
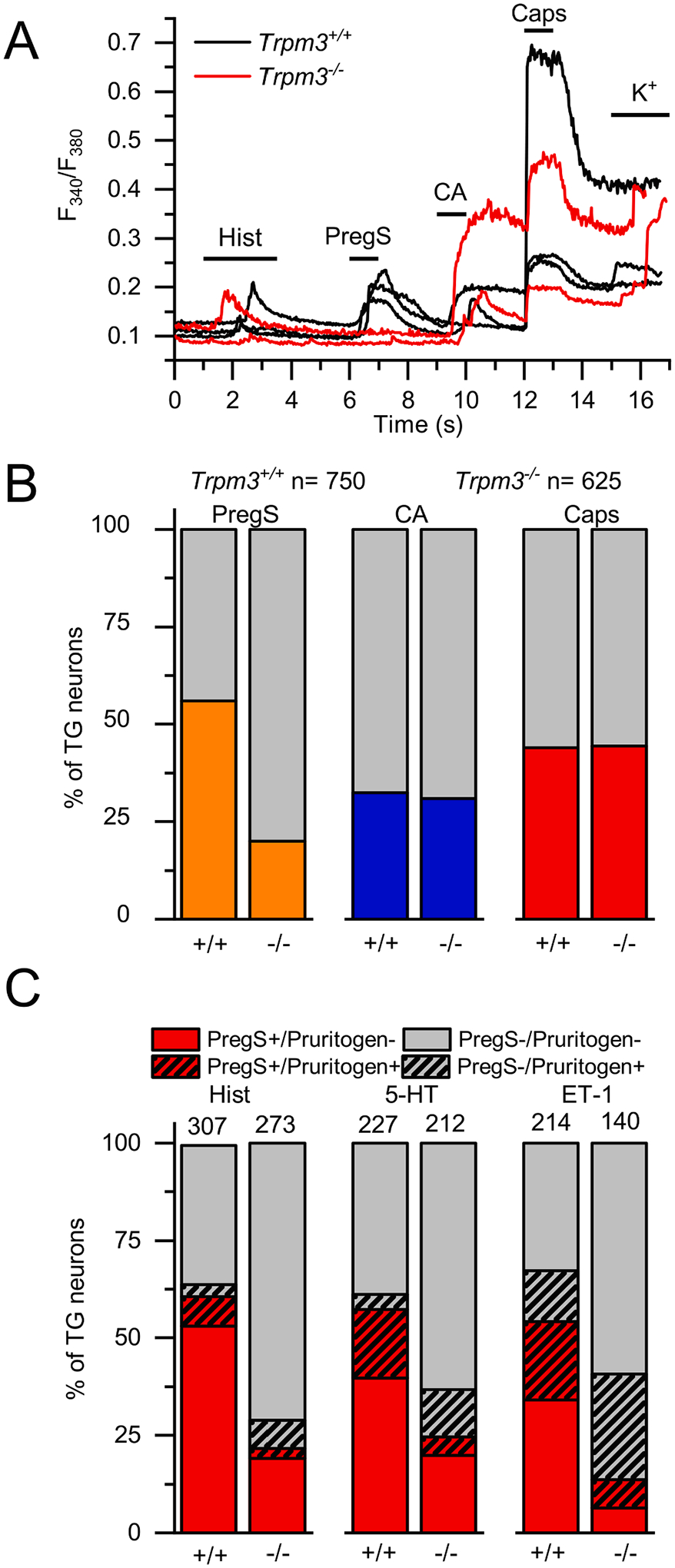
Effect of pruritogens in TG neurons isolated from *Trpm3*^+/+^ and *Trpm3*^−*/*−^ animals. (A) Representative traces showing typical changes in intracellular Ca^2+^ concentration of TG neurons isolated from *Trpm3*^+*/*+^ and *Trpm3*^−*/*−^ mice in response to 100 μM Hist, 20 μM PregS, 100 μM CA, 1 μM Caps as indicated in the panel. 25 mM KCl was used as positive control to depolarize the neuronal cell membrane. (B) Percentage of TG neurons form *Trpm3*^+*/*+^ and *Trpm3*^−*/*−^ mice responding to PregS (PregS+), CA (CA+) and Caps (Caps+). The measurements were carried out as in panel (A). (C) Percentage of TG neurons form *Trpm3*^+*/*+^ and *Trpm3*^−*/*−^ mice responding to 100 mM Hist, 100 mM 5-HT and 100 nM ET-1 in the experiments. Sample size (n) is indicated over the columns. The measurements were carried out as in panel (A). In each group, neurons were isolated from ≥3 mice, each measured in independent experiment.

Next, we tested whether cellular responses induced by pruritogens are related to TRPM3. For this purpose, we isolated somatosensory neurons from TGs of *Trpm3*^+*/*+^ and *Trpm3*^−*/*−^ mice and investigated *in vitro* cellular Ca^2+^ responses evoked by Hist, 5-HT, ET-1 and PregS, CA and Caps, agonists of TRPM3, TRPA1 and TRPV1, respectively. Different pruritogens were tested in individual experiments to avoid potential interactions. Only those cells were considered sensory neurons and included in the subsequent analysis which responded to depolarizing KCl solution or Caps applied at the end of the measurements, as shown in Fig. 3A. As expected, the ratio of PregS responsive (PregS+) neurons was strongly reduced in *Trpm3*^−*/*−^ TG neurons, although, consistent with previous results [32], some neurons still responded to PregS suggesting other, as yet unidentified targets available in *Trpm3*^−*/*−^ animals. The ratios of CA+ and Caps+ neurons were practically identical in the presence and absence of TRPM3 (Fig. 3B). Pruritogens activated a subpopulation of both PregS+ and PregS− neurons in *Trpm3*^+*/*+^ animals, indicating that the pruritogen-induced responses do not correlate with TRPM3 expression. Most importantly, the ratios of the neurons responding to Hist (10.7 vs. 9.9%; Х^2^ = 0.115, p = 0.735), 5-HT (21.6 vs. 17.0%; Х^2^ = 1.489, p = 0.222) and ET-1 (33.2 vs. 34.3%; Х^2^ = 0.006, p = 0.939) were not different between the *Trpm3*^+*/*+^ and *Trpm3*^−*/*−^ groups (Fig. 3C).

### 3.4. Pharmacological blockade of TRPM3 inhibited PregS evoked activation of trigeminal sensory neurons but did not affect pruritogen induced cellular responses

**Fig. 4.**
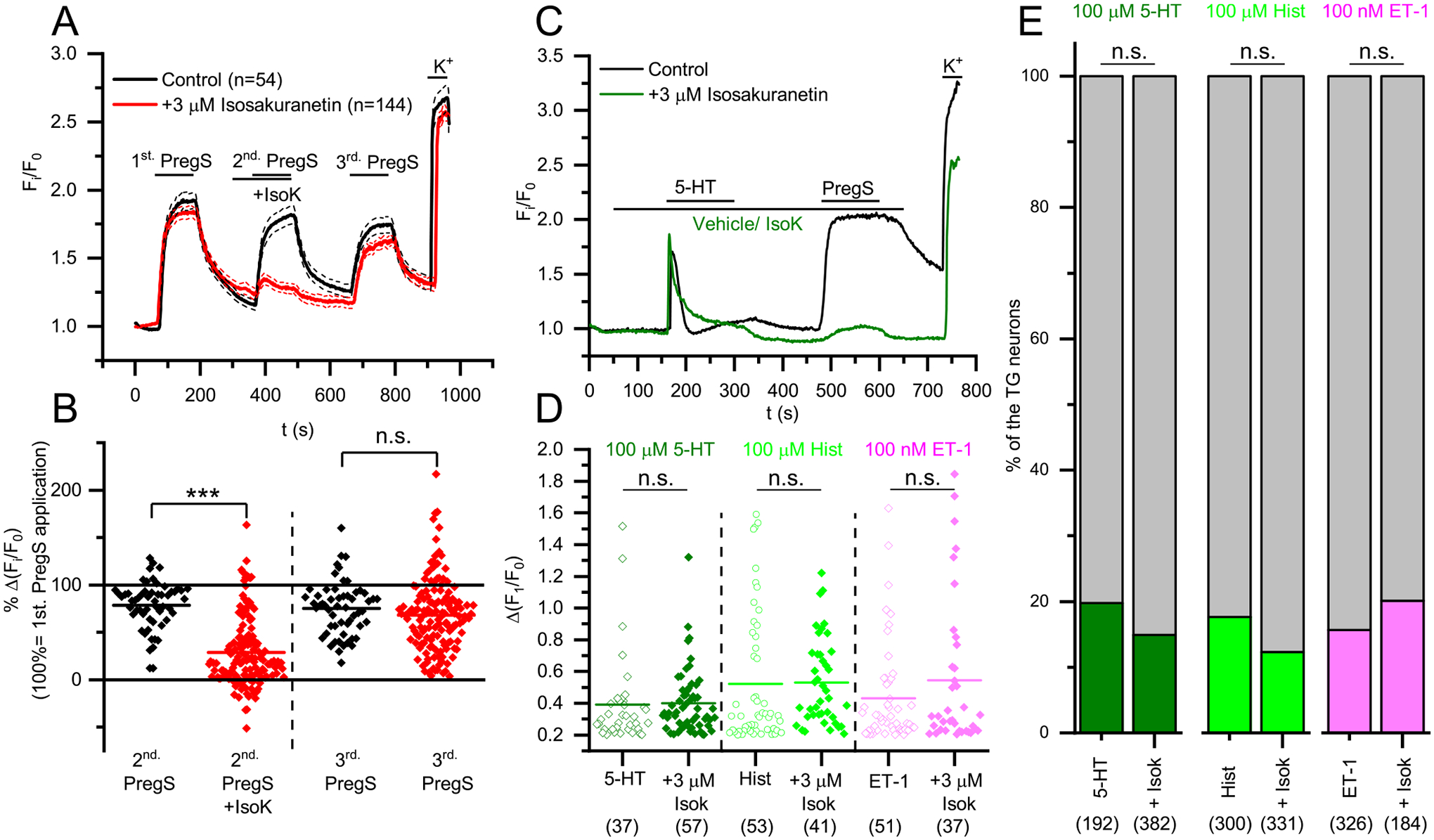
Effect of Isok on the Ca^2+^ signals evoked by PregS and pruritogens in *Trpm3*^+*/*+^ mice. (A) Averaged traces of several PregS+ TG neurons from *Trpm3*^+*/*+^ mice showing mean changes in intracellular Ca^2+^ concentration in response to 20 μM PregS in the presence or absence of 3 μM IsoK. 25 mM KCl was used as positive control to depolarize the neuronal cell membrane. (B) Statistical analysis of Isok effect on PregS induced Ca^2+^ transients in PregS+ *Trpm3*^+*/*+^ TG sensory neurons as shown in panel (A). Values are given as percentage of the first PregS-induced Ca^2+^ transient. Dots represent individual neurons, and horizontal lines indicate mean values. Effect of Isok was compared to the vehicle treated control group by Mann-Whitney test, n.s: p > 0.05 (non-significant), ***p < 0.001. (C) Representative traces showing typical changes of intracellular Ca^2+^ concentration in TG neurons from *Trpm3*^+*/*+^mice in response to 100 μM serotonin and 20 μM PregS in the presence and absence of 3 μM Isok. 25 mM KCl was used as positive control to depolarize the neuronal cell membrane. (D) Statistical analysis of Isok effect on the pruritogens induced Ca^2+^ transients in pruritogen responsive TG sensory neurons from *Trpm3*^+*/*+^ animals. Values are given as Δ(F1/F0), dots represent individual neurons, and horizontal lines indicate mean values. Effect of Isok was compared to the vehicle treated control group by Mann-Whitney test, n.s: p > 0.05 (non-significant). (E) Percentage of TG neurons form WT mice responding to Hist, 5-HT and ET-1 in the presence and absence of 3 μM Isok. The measurements were carried out as in panel (C). The distribution of the pruritogen responders among TG sensory neurons was compared using Chi squared test, n.s.: p > 0.05 (non-significant). Responders are marked with the indicated colors and non-responders are marked with grey. In each group, neurons were isolated from ≥3 mice and measured in independent experiments.

Finally, we investigated how the pharmacological blockade of TRPM3 influences cellular activation of sensory neurons isolated from TGs of *Trpm3*^+*/*+^ animals. TRPM3 agonist PregS, as well as Hist, 5-HT, and ET-1 were applied during intracellular Ca^2+^ measurements in the presence and absence of the TRPM3 antagonist Isok (Fig. 4). As shown earlier on DRG derived neurons [45], PregS-induced responses were strongly inhibited by Isok in a reversible way (Fig. 4A–B). In contrast, pharmacological inhibition of TRPM3 did not affect the neural activation induced by the endogenous pruritogens: neither the amplitude of the pruritogen-induced Ca^2+^ signals nor the ratio of the Hist+, 5-HT+ and ET-1+ neurons were significantly changed in the presence of 3 μM Isok (Fig. 4C–E).

## 4. Discussion

Emerging evidence suggests that pruriceptive neurons form a subpopulation within nociceptive neurons, rather than forming a purely pruritogen-specific peripheral sensory neuron population, [46–49] but the organization of nociceptive and pruriceptive sensory system is still unclear. Non-pruritogenic nociceptive neurons were identified to be unresponsive to pruritic chemical signals [2], and there are numerous attempts to identify itch-specific molecular markers. Such studies not only aim at identifying itch-specific/selective neurons and pathways but are also motivated by the medical need to identify molecular targets for pharmacotherapies selectively alleviating itch or pain [2,6,41,48,50,51]. TRP channels are long-chased targets for analgesic therapies but they seem to be promising targets in the management of pruritus, as well [10,52–54]. Among TRP channels, the thermosensitive TRPV1 and TRPA1 are of special importance: they seem to be promiscuously expressed in nociceptive and pruriceptive neurons and were shown to play role in the sensory transduction of both pain and itch.

Moreover, other thermosensitive TRP channels can be also involved in the development of both itch and pain. TRPV4 and TRPV3 are expressed in non-neuronal cells of the skin, and can play roles in the release of endogenous pruritogens and algogens, especially related to inflammation [55–59]. The role of TRPV4 was described in both allergic and non-allergic pruritus by mediating 5-HT release from mast cells and keratinocytes, respectively [60], and its activation in keratinocytes results in ET-1 release, as well, which is thought to play a role in sunburn-associated pain [61]. Beyond 5-HT release, TRPV4 is also involved in the sensory transduction of 5-HT-mediated itch in the pruriceptive fibers [62], and as an osmo-mechanoreceptor it plays a role in the development of mechanical hyperalgesia [63–66]. TRPV3 is also highly expressed by keratinocytes and its activation can contribute to inflammation and several forms of itch by inducing the release of inflammatory and pruritic mediators [58,67–70], similar to non-neuronal TRPV4.

TRPM3 is also a thermosensitive nociceptor ion channel activated by noxious heat and the endogenous neurosteroid PregS. Its selective activation results in neuropeptide release from the sensory terminals and evokes nociception in rodents [30,32]. Certain ligands and ligand combinations open an extra permeability pathway in the channel, which results in a strong depolarizing current at negative membrane potentials and in the exacerbation of pain sensation, as well [30,37]. In the nociceptive system, TRPM3 functions seem to partially overlap with other thermosensitive TRP channels. Together with TRPV1 and TRPA1, TRPM3 plays a crucial role in the sensory transduction of noxious heat sensation and it was also found to be involved in inflammatory heat hyperalgesia. Moreover, beyond the functional similarities, its expression in the somatosensory neurons of DRGs largely overlaps with TRPA1 and TRPV1 [32,38]. The functional and anatomical overlap, and the molecular relationship between these thermosensitive TRP channels led to the plausible preposition that TRPM3 expressed by the somatosensory neurons could share even more functions with TRPV1 and TRPA1, for example in pruriception.

As mentioned above, beyond nociception and thermosensation, TRPV1 and TRPA1 are also important players in itch transduction at the sensory terminals: they were shown to be involved in the detection of various forms of itch (for a recent review see [9]). For example, TRPV1 was found to be involved in histamine receptor and protease activated receptor 2 (PAR2) signaling [31] and TRPA1 was shown to transmit the pruritic effect of 5-HT [71], bile acid [34], activators of Mas-Related G Protein–Coupled Receptors (Mrgprs) [35], and thymic stromal lymphopoietin (TSLP) [72].

In our study, we challenged the pruriceptive role of TRPM3 for the first time and we investigated its selective role in itch and nociception using the generally accepted cheek model [44,49]. Our results clearly indicate that TRPM3 is involved in nociception but not in pruriception.

The role of TRPM3 in thermal nociception and inflammatory heat hyperalgesia is well established by previous results [32,37,38,73], and TRPM3 antagonists evoked promising analgesic effects in animal studies [45,74,75]. Our *in vivo* results obtained in the cheek model further support the previous conclusion, and demonstrate that the selective pharmacological activation of TRPM3 resulted in nociception even in the cheek, i.e., in the innervation area of TGs, and this was abolished by the genetic ablation of the channel. As opposed to nociception, TRPM3 agonists did not induce scratching behavior in the animals, suggesting that TRPM3 activation did not evoke itch sensation on its own. Although its general activation resulted exclusively in pain, these findings do not exclude that expressed in certain subpopulation of the somatosensory neurons, TRPM3 can contribute to pruritic signaling and take part in the transduction of itch. For example, it is also known from TRPV1 that its general activation by Caps evokes a burning pain but not itch sensation [44]. However, as discussed above, TRPV1 is expressed in pruriceptive nociceptors, and takes part in the transduction of histaminergic, and non-histaminergic itch [31], as well. Furthermore, if TRPV1 is expressed exclusively in pruriceptive, MrgprA3 expressing neurons, its activation with Caps results in itch but not pain sensation [41]. Therefore, we tested whether TRPM3 is necessary for the pruriceptive effect of highly relevant endogenous pruritic mediators, Hist, 5-HT and ET-1. We found that each mediator evoked similarly intense scratching in *Trpm3*^+*/*+^ and *Trpm3*^−*/*−^ mice injected to either the cheek or the nape, skin areas that are innervated by neurons from the TG and the DRGs, respectively. In good accordance with the *in vivo* findings, the ratio of the trigeminal sensory neurons activated by Hist, 5-HT, and ET-1 was not affected by the deletion of *Trpm3*, although the investigated pruritogens activated both TRPM3 expressing (PregS+) and TRPM3 non-expressing (PregS−) neurons of wild type (*Trpm3*^+*/*+^) animals. Moreover, the pharmacological blockade of TRPM3 by Isok affected neither the number of pruritogen responsive neurons nor the amplitude of their Ca^2+^ transients evoked by the itch mediators. These results strongly argue for that TRPM3 does not play any significant role in cellular signaling events evoked by Hist, 5-HT or ET-1 that result in the pruritic effect of these compounds.

Intriguingly, ET-1 induced more intense scratching in *Trpm3*^−*/*−^ animals. This finding may be explained by the common observation and experimental findings that painful stimuli inhibit itch [76,77]. In the last decade, the underlying spinal circuits were also revealed involving vesicular glutamate transporter type 2 (Vglut2) expressing nociceptors [4,5], Bhlhb5 transcription factor expressing inhibitory interneurons and kappa opioid receptor signaling [78,79] (for a current review see [80]). Regarding our results, it is possible that the lack of TRPM3 results in decreased (basal) activity of the nociceptive neurons, which consequently leads to enhanced itch signaling in certain cases. It cannot be excluded that ET-1 itself causes a minor activation of the nociceptors which partially inhibit itch responses, but this inhibition is diminished in *Trpm3*^−*/*−^ animals. ET-1 was also reported to mediate nociception [81,82], although in our experiments it initiated only moderate nocifensive behavior (wiping) and was characterized by high scratch ratio as a mainly pruritogenic substance.

Our results led to the conclusion that TRPM3 is exclusively related to nociception but not itch transmission, since it was not involved in the transmission of the pruritic effect of key endogenous itch mediators (i.e., Hist, 5-HT, and ET-1). However, it cannot be excluded that TRPM3 might be necessary for itch evoked by some other mechanisms.

The effect of temperature changes on itch is controversial. Noxious heat (as other noxious stimuli) are known to inhibit itch [80], but moderate warming can amplify pruritus, as reported especially in atopic dermatitis [83,84]. However, the role of TRPM3 is less likely in warm induced atopic pruritus. Although chemical activation of TRPM3 is already potentiated at 33 °C, its heat-induced activation is more prominent at noxious temperatures [32]. Compared to TRPV1, the current-temperature relationship curve of TRPM3 is shifted slightly towards higher temperatures [85]. In line with this characteristic, behavioral experiments indicated that TRPM3 plays an essential role in noxious heat sensation, but also that its genetic ablation had only a moderate effect on warm sensation in neutral temperature zone [32,38,86]. A recent study described that warm induced pruritus and pruritogen release from atopic keratinocytes is mediated by TRPV3 [68].

Our results further support the idea that TRPM3 represents a promising candidate target to specifically treat pain. Earlier results already showed that its genetic ablation or pharmacological inhibition alleviates chemical and thermal nociception, as well as inflammatory pain in the innervation area of DRGs [32,45,73,87]. Our results also demonstrated that PregS- or CIM0216- induced nociception is diminished in *Trpm3*^−*/*−^ animals in the cheek model, in the trigeminal innervation area, as well. Moreover, we also demonstrated that it does not take part in itch sensation and its inhibition or deletion hardly influence pruritic responses. With the previous results, our findings suggest that TRPM3 may be a superior target in pain therapies than other TRP channels, including the long pursued TRPV1. Indeed, activation or inhibition of TRPV1 can drastically influence core body temperature, which was not found in case of TRPM3 [32,88–90], and our results suggest that the role of TRPM3 is more selective for nociception over pruriception than TRPV1 or TRPA1. Moreover, this latest result can have an impact on the better understanding of the molecular organization of nociceptive and pruriceptive systems.

As for most animal studies, it is important to consider to what extent we can translate these results to humans. In general, the cheek model can similarly discriminate between itch and nociception as subjective reporting of human subjects [44] and the applied Hist, 5-HT, and ET-1 are known to induce itch both in mice and humans [9,43,91,92]. Although, based on our best knowledge, effects of TRPM3 ligands were not published in human *in vivo* studies yet, the available pharmacological and cellular data suggest that the mouse and human wild type TRPM3 are functionally identical: they share agonists, antagonists, and regulation by phospholipids, as well as by βγ subunits of G proteins [87,93–96]. These data suggest that selective targeting of nociception via TRPM3 may be a promising approach even in human analgesia.

## Supplementary Material

Supplemental Video-1

Supplemental Video-2
